# The Trap of the COVID-19 Pandemic: Italian Adolescents Fare Well at First, Maybe Thanks to Protective Trait Expression

**DOI:** 10.3390/children9111631

**Published:** 2022-10-26

**Authors:** Anna Malerba, Sara Iannattone, Giorgia Casano, Marco Lauriola, Gioia Bottesi

**Affiliations:** 1 Department of General Psychology, University of Padova, 35131 Padova, Italy; 2 Department of Developmental and Social Psychology, Sapienza University of Rome, 00185 Rome, Italy

**Keywords:** COVID-19, positivity, intolerance of uncertainty, psychopathological symptoms, psychological adjustment, adolescence, longitudinal study

## Abstract

Abundant research indicates that the COVID-19 pandemic has been negatively affecting mental health in adolescence. Few works, however, benefit from data from the same sample before and after the onset of the pandemic. The present longitudinal study involved a non-clinical group of 136 Italian adolescents (M_age_ = 16.3 years ± 1.08, 67% girls) to investigate their psychological response to the first lockdown and explore the role of a protective trait (i.e., Positivity) in moderating the effect of Intolerance of Uncertainty (IU) on internalizing symptoms before and during the COVID-19 outbreak. Participants completed self-report questionnaires assessing psychopathological symptoms, psychological well-being, IU, and Positivity on three separate occasions: October 2019 (T1), January 2020 (T2), and April 2020 (T3). The results showed that internalizing and externalizing symptoms as well as psychological well-being did not vary significantly over time. Positivity was found to significantly moderate the relationship between IU and internalizing symptoms at T3 (i.e., during the COVID-19 lockdown) only. Overall, our findings suggest that the teenagers’ good adjustment to the initial phase of the pandemic might have been associated with the enhanced weight of the Positivity trait, which may have encouraged a positive attitude towards self, life, and the future.

## 1. Introduction

In February 2020, Italy was struck by the spread of a new strain of coronavirus, the SARS-CoV-2, which caused the COVID-19 infectious disease pandemic in the following months. The need to limit the contagion warranted prompt action by the national administration resulting in a total lockdown, whereby the entire population was conditioned to restrictive physical and social distancing measures, substantially limiting their movements and their engagement in social activities. Restrictions became effective as early as the 11th of March and lasted until the 3rd of May 2020 (so-called “phase 1”) [1]. Generally speaking, the COVID-19 outbreak had severe repercussions on mental health and well-being worldwide, leading to a sharp increase in cases of major depressive disorder and anxiety disorders in the adult population [2]. The greatest increases were found in places highly affected by the pandemic, i.e., places with the highest daily COVID-19 infection rates and the greatest reductions in human mobility. Indeed, Italy experienced a 21 to 25% change in prevalence rates of anxiety disorders and major depressive disorder in the first year of the pandemic outbreak [3].

In this scenario, people in developmental age should be carefully taken into consideration. The COVID-19 lockdown was characterized by the mandatory closure of schools. As a consequence, youth were subject to a major and long-lasting disruption in their daily lives: a structured program of at-home remote classes was put into place for all students, setting off widespread uncertainty around their immediate and mid-term future [4]. A new set of challenges emerged: exposure to physical social spaces was drastically reduced and virtual ones became prominent to many [5], learning modalities changed radically, and exposure to the family environment suddenly increased [6]. Moreover, exposure to once pleasurable activities (e.g., physical activity, social engagements, travelling) decreased, while fear of contagion was being encouraged both by government agencies and media outlets [7]. The exposure to such environmental changes together with major reductions in regulatory and coping mechanisms to face them [4] was expected to have substantial effects especially on internalizing symptoms in children and adolescents [8]. Importantly, adolescence is known as a developmental period marked by rapid biological and social changes [9]. It is connected to specific developmental tasks such as the definition of personal identity through the development of autonomy and bonds with peers [10,11]. The risk of interference of the described pandemic-related factors with developmental trajectories led authors to speculate that youth might be at an even greater risk of adverse mental health outcomes from infectious outbreaks [12,13], also considering that adolescence is *per se* characterized by a high psychopathological vulnerability, especially in terms of internalizing problems [14].

Nonetheless, estimates seem rather inconclusive in evaluating early internalizing outcomes for the adolescent population [2]. The World Health Organization (WHO) indicates that a major limitation of existing estimates comes from the scarcity of baseline pre-pandemic comparison data to match measurements collected during the first months of this unanticipated event. A systematic review and meta-analysis of longitudinal cohort studies examining changes in mental health among the same group of children and adolescents (*n* = 38) before vs. during the pandemic in 2020 found no significant differences [15]. Two meta-analyses of pooled prevalence rates (from 2020), on the other hand, were convergent in reporting increased rates of depression and anxiety during COVID-19, particularly for older adolescents, especially girls, with increasing estimates over time [16,17].

To date, published literature on mental health outcomes of adolescents during the pandemic is wanting, especially in the Italian context [18]. In this regard, two well-known risk and protective factors of internalizing outcomes worthy of further attention are, respectively, Intolerance of Uncertainty (IU) and Positivity [19,20,21].

### 1.1. Intolerance of Uncertainty

Uncertainty was plainly described as ‘the psychological state of “not knowing”’ [22] (p. 199), and, more specifically, as the perceived lack of sufficient or salient information in a given situation [23]. It is a characteristically unpleasant state that can arise in several circumstances. IU, distinctively, is the stable disposition not to tolerate the aversive reactions elicited by uncertainty, maintained by the perception of uncertainty itself [23].

Originally conceptualized as the key cognitive vulnerability factor for worry [24], IU is now established as a trans-diagnostic risk factor across internalizing disorders, putatively underlying neuroticism [23,25]. Indeed, Carleton has described IU as a transdiagnostic dispositional risk factor for clinically relevant anxiety and depression. Consistently, IU has been shown to account for statistically significant amounts of variance in symptoms of several anxiety disorders and to be strongly associated with depression (for a review see Carleton and colleagues [23]) [26].

To date, few studies have investigated IU in adolescent samples, although this developmental stage seems per se characterized by elevated uncertainty, thus exposing teenagers to a higher risk of developing psychopathological symptoms [27]. Some studies have uncovered the role played by IU in several disorders in adolescence [14], although research in this field is still in its infancy. Research on worry in adolescence showed that around this age concerns grow increasingly abstract, detailed, and related to temporally distant factors, thus proving that adolescents, much like adults, can suffer from concerns surrounding the future in general, in addition to those surrounding social evaluation or academic performance [14,28].

A 5-year, ten-wave longitudinal study of 338 high school adolescents provided enticing insight into the evolution of IU in this population. Despite observing relative stability of change trajectories in IU, the main findings showed that the highest levels of IU were observed at the beginning and end of secondary school (i.e., time points 1 and 10). The authors argued these to be transition periods, marked by significant and simultaneous changes in both internal and external domains of adolescent life, explaining temporary yet meaningful inflations in IU. This evidence seems to support the idea that IU is also a malleable construct, since prolonged periods during which adolescents are challenged by uncertainty can cause alterations in this usually stable disposition [28].

According to the WHO [29], extended school closures have left young people “vulnerable to social isolation and disconnectedness which can fuel feelings of anxiety, uncertainty, and loneliness and lead to affective and behavioral problems” (p. 20). Indeed, uncertainty has afflicted youth during the COVID-19 pandemic, as young people have experienced it from when evaluating the probability of infecting family members to when anticipating educational and employment outcomes in the future, following several months of school closure [4]. When adolescents catastrophically misinterpret uncertainties such as those naturally occurring within a pandemic [30,31], IU is likely to grow, and this represents a breeding ground for adverse outcomes such as anxiety and depression [32]. Extant research has chiefly examined the role of IU in buffering health-related anxiety in adults during COVID-19 [32], while, to our knowledge, no study has yet tapped into the relationship between adolescent IU and internalizing symptoms during this pandemic.

### 1.2. Positivity

In 2004, the WHO published an influential report addressing the promotion of mental health and redefined it as: not merely the absence of illness, but the presence of “a state of well-being in which the individual realizes his or her abilities, can cope with the normal stresses of life, can work productively and fruitfully, and is able to make a contribution to his or her community” [33] (p. 12). This contribution brought about the rejection of the traditional conceptualization of well-being and ill-being as two extremes of a continuum, thus allowing for the recognition of mental health as a unique set of “symptoms of positive functioning”, as opposed to the constellation of symptoms of bad functioning involved in mental illness [34].

Building upon the call for research on adequate indicators of positive mental functioning or flourishing [35], Caprara and colleagues developed a conceptualization of the cognitive component to well-being encompassing self-esteem [36], life satisfaction [37], and dispositional optimism [38]. These dimensions represent phenomenological expressions of the latent construct named Positivity [39]. Positivity is described as a “pervasive mode of appraising, viewing, and perceiving life from a positive stance” [40] (p. 353), a process closely associated with the management and consequences of positive affect. Positivity predisposes people to “recruit, amplify and benefit from it through the promotion of experiences that carry positive feelings and by enhancing and prolonging their savoring” [41] (p. 4).

Extant literature underscores the heritable mechanism behind individual positive orientation [39], while also revealing a meaningful susceptibility of this disposition to environmental influence [42]. A significant component linked to experience speaks to its moderate malleability: life experiences play a role in the different forms that self-esteem, life satisfaction and optimism may take and in their combinations [41]. For example, a daily study on an adolescent sample has expounded on the genetic evidence, showing a significant variation of Positivity from one day to the next [43], while evidence from a longitudinal study showed a high degree of consistency of positive orientation over adolescents’ developmental trajectory [44].

Several studies have provided evidence supporting the role of Positivity as an individual’s resource sustaining optimal functioning in several domains (e.g., [45,46]) and empirical studies have found significant positive links between Positivity and different indices of well-being in adolescents and young adults (e.g., [34,40,47]). Notably, two studies have pointed out its key contribution to predicting adolescent and adult resilience, which is the ability to adapt successfully to changing and stressful environmental challenges and life events [48,49]. Specifically, Milioni and colleagues [48] provided longitudinal evidence for the predictive effect of Positivity on later adolescent ego-resiliency over 10 years, while the contrary was not found, and a study [49] conducted during the COVID-19 pandemic corroborated the protective role of Positivity in reducing adults’ anxiety and depressive symptoms.

Finally, Positivity is consistently linked to lower levels of internalizing and externalizing symptoms in early adolescents [19,50]. Extant literature emphasizes the possibility that Positivity might constitute a “syndrome of optimal functioning”, acting as a protective factor in general against mental illness and specifically against depression [39]. This beneficial effect is posited in accordance with cognitive theories that see negative views of the self, the world, and the future as the basis of depression [39,51].

### 1.3. The Present Study

The COVID-19 pandemic has acted as a major stressor on adolescents’ psychological well-being [2], requiring them to tolerate an uncertain future drawing upon personal resources to face a pervasive uneasy mental state [4,52]. However, research addressing this issue is constrained by the limited number of studies providing same-sample data on mental health both pre- and during the COVID-19 pandemic [2,15]. Furthermore, to our knowledge, only one international study [53] has navigated the role played by stable, trait-like dispositions in moderating mental health outcomes during such uncertain and unsettling times for youth [49]. However, such an attempt is yet to be made in Italy.

Extant literature holds Positivity and IU as key cognitive precursors to internalizing problems (i.e., anxiety and depressive symptoms) [19,23], the former acting as a protective factor providing the dispositional base to experience happiness in life and the latter hindering adolescents’ capacity to endure uncertainty, thus promoting emotional distress (e.g., [54]). Although both constructs have been defined as stable dispositions, some studies highlighted their malleability under the influence of (stressful) environmental factors [28,39]. Nevertheless, to the best of our knowledge, no study has yet examined IU in an adolescent sample during the COVID-19 pandemic. Given the established and strong link between IU and anxiety and depressive symptoms [23], an important step is to investigate its role in adolescence during the COVID-19 pandemic, in light of the aforementioned factors: pandemics in general are ridden with uncertainty [30], adolescence is itself characterized by elevated uncertainty [27], and internalizing problems often emerge in adolescence [14]. Furthermore, no study has yet investigated the relationship between IU and Positivity in affecting internalizing symptoms during any time. The relevance of providing further evidence on protective and risk factors to adolescent mental health in trying times lies in the opportunity to identify useful indicators of resilience or, conversely, vulnerability, thus contributing to the implementation of broad-spectrum and trait-specific intervention programs [39].

#### Aims and Hypotheses

In light of the aforementioned considerations, we involved an Italian non-clinical adolescent sample with the twofold aim of:

1. Describing what changes, if any, have occurred in internalizing and externalizing symptoms, and psychological well-being with the first COVID-19 lockdown. We were particularly interested in investigating those psychopathological dimensions that are chiefly associated with the social and scholastic environment (i.e., attention problems, social, scholastic and separation anxiety).
  According to the previous literature, we hypothesized to observe a substantial stagnation in internalizing (H1a), externalizing (H1b), and attention problems (H1c), social anxiety (H1d), and generalized anxiety (H1e) [15,55]. No studies conducted on non-clinical adolescent samples explored these issues in relation to separation anxiety, school anxiety, and psychological well-being, thus we did not make specific predictions.
2. Exploring whether Positivity moderated the relationship between IU and internalizing symptoms before and during the COVID-19 lockdown. We chose to focus on internalizing symptoms in light of the literature supporting their association with both IU and Positivity [19,23,26].
  In particular, we hypothesized that IU would positively predict internalizing symptoms at both times (H2) [23,26], and Positivity would negatively predict internalizing symptoms at both times (H3) [19]. Moreover, we also expected sex (H4) [56,57] and age (H5) [58,59] to significantly predict internalizing symptoms at both times, with higher levels more likely in girls and older adolescents.
  We chose to test the same model twice, including the same variables but at different time points, because we had the unique opportunity of comparing the moderating effect of a trait-like disposition before and during the lockdown to observe the lockdown’s effect on trait-like dispositions’ interactions; this enabled us to scrutinize whether and how a simple cognitive and psychopathological system reacts to a major stressor, capitalizing on cognitive resources to produce resilience. This condition offered a unique standpoint that enabled us to investigate the effect of the environment on two cognitive factors that, albeit dispositional, can take different forms and express differently under certain circumstances [28,39,41]. No previous research has focused on this issue, so we did not formulate any specific hypothesis.

## 2. Materials and Methods

### 2.1. Participants and Procedure

The present work stems from a larger longitudinal study originally designed to investigate the role of protective factors for psychopathology in nonclinical adolescents across six months. Participants were recruited at high schools in Veneto (one of the most severely affected regions of Italy from COVID-19-related mortality [60]). After obtaining the approval of the school directors, a written informed consent form was collected from parents or legal guardians of minor students, whereas 18 years old students provided their own consent. The study was conducted in accordance with the Declaration of Helsinki and approved by the local Ethical Committee.

An online survey was developed in the Google Modules platform. It included a battery of self-report questionnaires measuring several constructs (e.g., happiness, mindfulness, broadband symptomatology) and a socio-demographic survey, soliciting information on sex, age, school year, and presence of psychological difficulties. Given the aims of the current study, we only considered the questionnaires described in Section 2.2. The first two administrations took place in October 2019 (T1) and January 2020 (T2) at the school’s computer room. Completion required 45–50 min on average and occurred during school hours. The third administration (T3) occurred during the national lockdown (i.e., April 2020), when schools were closed to prevent contagion. Consequently, the form was delivered to participants through the school’s web platform and completed outside the school building. Data collected at each time point were paired through alphanumeric codes, assigned to each participant.

The final sample consisted of 136 Italian adolescents (M_age_ = 16.3 years, *SD* = 1.08), including 44 boys (32.4%) and 92 girls (67.6%) aged 14 to 18. 2.9% of the sample was 14 years old, 22.8% was 15, 32.4% was 16, 25.7% was 17 and 16.2% was 18 years old. 9.6% of participants answered yes to a question addressing having had, either currently or in the past, psychological difficulties that warranted professional help (e.g., problems with anxiety and depressive symptoms).

### 2.2. Measures

The Positivity scale (P Scale; [19]) evaluates the respondents’ dispositional tendency to have a positive attitude towards themselves, their lives, and their future. The scale is composed of 8 items rated on a 5-point Likert scale ranging from 1 (strongly disagree) to 5 (strongly agree). The P Scale operationalizes Positivity in three aspects (i.e., self-esteem, life satisfaction, and optimism); however, there is consensus that these facets can be traced to a single, general self-evaluative latent construct. Higher scores reflect greater Positivity. The unidimensionality of the questionnaire was confirmed both in adult and adolescent samples [19,50,61]. The P Scale showed high reliability coefficients in secondary school students (ω = 0.84) [19].

The Intolerance of Uncertainty Scale-Revised (IUS-R; [27]) is a 12-item self-report questionnaire assessing the disposition not to tolerate the aversive reactions elicited by uncertainty. Each item is rated on a 5-point Likert scale (1 = Not at all like me, 5 = Entirely like me). The Italian version of this measure showed excellent internal consistency in a large adolescent sample (Cronbach’s α = 0.90, McDonald’s ω = 0.90) and a good one-month test-retest reliability (r = 0.74) in undergraduate and adult samples [62]. Higher scores reflect higher levels of IU.

The Youth Self-Report 11–18 (YSR; [63,64]) is a self-report questionnaire consisting of 113 items that examine social competencies and psychopathological behavior. The latter includes 9 syndrome scales: anxious/depressed; withdrawn/depressed; somatic complaints; social problems; thought disorders; attention disorders; deviant behavior; aggressive behavior and other problems. These subscales are then grouped to obtain three global scales for internalizing problems, externalizing problems, and total problems. In our study, we considered two global scales (internalizing problems and externalizing problems), and one syndrome scale (i.e., attention problems). Items of the internalizing problems global scale reflect anxiety and depression symptoms (e.g., fears, worries, isolation, sadness, crying a lot). The externalizing problems global scale refers to aggressive and rule-breaking behavior (e.g., lack of guilt, substance use, mean and disobedient behavior). The internalizing problems scale includes the social withdrawal, somatic complaints, and anxiety–depression scales. The externalizing problems scale includes the deviant behavior and aggressive behavior scales. Higher scores on each scale reflect higher levels of the measured syndrome or syndromes. This tool has good reliability, with Cronbach’s alpha ranging from 0.71 to 0.95.

The Self Administrated Psychiatric Scales for Children and Adolescents-Anxiety evaluation scale (SAFA-A; [65]) is an Italian self-report questionnaire for the evaluation of anxiety symptomatology in the adolescent population ranging between 11 and 18 years old. This tool measures generalized anxiety, social anxiety, separation anxiety, and school-related anxiety. Respondents provide answers to 50 items, among three alternatives: true, partly true, and false. This tool showed a good test-retest reliability in its original validation (r > 0.75), and a very good internal consistency (Cronbach’s α coefficient > 0.85) [65].

The Psychological Well-Being Scales (PWB; [66,67]) is an 18-item self-report questionnaire that evaluates six dimensions of psychological well-being, by Ryff’s and Keyes’ theoretical model: self-acceptance, autonomy, environmental mastery, personal growth, purpose in life, and positive relations. Participants provide their responses on a six-point Likert scale (1 = strongly disagree, 6 = strongly agree). The factorial structure of the PWB has been also supported among Italian adolescents and showed adequate psychometric properties (Cronbach’s α ranging from 0.60 to 0.70) [68,69]. For the present study, we only considered the PWB total score, which showed a good internal consistency (Cronbach’s α = 0.86).

### 2.3. Data Analysis

First, we calculated descriptive statistics to highlight the main features of the population under study.

Then, a series of 1 × 3 repeated measures analyses of covariance (ANCOVAs) were performed to examine changes over time (T1 vs. T2 vs. T3) in the following variables: YSR externalizing problems, internalizing problems, and attention problems scales; SAFA-A generalized anxiety, school-related anxiety, social anxiety, and separation anxiety scales; PWB total score. In all analyses, age and sex (male = 1, female = 2) were included as covariates since, in light of existing literature [56,57,58,59], they were expected to have an important influence on the primary variables.

Subsequently, Pearson’s bivariate correlations between the YSR internalizing problems scale (i.e., outcome variable of the following analysis), and the scales assessing transdiagnostic risk and protective factors (i.e., IUS-R and P Scale) at T1 (i.e., pre-COVID-19) and at T3 (i.e., during COVID-19) were preliminarily run.

**Figure 1:**
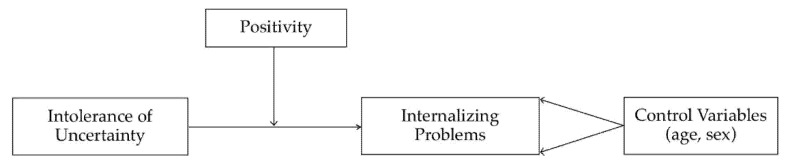
Proposed theoretical model.

Finally, two moderation models were tested (see Figure 1). In the first model, the YSR internalizing problems scale (T1) was included as the dependent variable, and the IUS-R (T1) and P Scale (T1) were included as predictors. The second model was the same but included scores on these three questionnaires (e.g., IUS-R, P Scale, and YSR internalizing problems) administered at T3. In both models, age and sex were included as covariates. In the first step of each model, the IUS-R was entered as a predictor of the YSR internalizing problems scale; the P Scale was then entered as an additional predictor in the second step, and finally, to test the interaction between the two predictors, the product of centered IUS-R and centered P Scale scores was entered as an additional predictor in the third step. Finally, to further describe the quality of significant interactions, we employed the Johnson-Neymann (JN) technique [70]. The JN technique is a tool for probing significant interactions, identifying values in the range of the moderator variable where the conditional effect of the predictor on the outcome transitions between statistical significance and non-significance. In this way, one can find the value of the moderator for which the ratio of the conditional effect (of the predictor) to its standard error is equal to the critical *t* score. Effectively, it identifies a “region of significance” of the effect of the predictor on the outcome, that being the range of values where the moderator acts as such on the predictor-outcome relationship. A bias-corrected bootstrapping method was applied for testing significant effects with 5000 bootstrap samples and 95% confidence intervals.

The statistical software JASP 0.16.3 [71] was used to run the ANCOVAs and bivariate correlations, while Model 1 of the SPSS PROCESS macro [70] was adopted to conduct moderation analyses. Statistical significance was set at *p* < 0.05.

## 3. Results

### 3.1. Changes in Psychopathological Symptoms and Psychological Well-Being over Time

When repeated measures ANCOVAs were computed, no change over time came to light in any of the considered scales (Table 1). No significant effects of time by sex or age emerged for these analyses (all *p*-values > 0.05).

### 3.2. Moderation by Positivity on the Relationship between IU and Internalizing Problems Pre and during COVID-19

Zero-order correlations (Table A1) showed that the IUS-R was positively associated with the internalizing problems scale of the YSR at both T1 and T3 (T1: *r* = 0.519, *p* < 0.001; T3: *r* = 0.408, *p* < 0.001). Along the same line, the P Scale was negatively correlated with the YSR internalizing problems scale at both time points (T1: *r* = −0.479, *p* < 0.001; T3: *r* = −0.295, *p* < 0.001).

The results of the first moderation model (T1, October 2019) are reported in Table 2. Entering the IUS-R in the first step as predictor of the YSR internalizing problems scale yielded a significant positive effect (*t* = 6.698, *p* < 0.001, *β* = 0.495, Adjusted *R*^2^ = 0.280 *ƒ*^2^ = 0.42). When the P Scale score was entered as an additional predictor in the second step, it showed a significant negative effect over and above the effects of the IUS-R (*t* = −5.903, *p* < 0.001, *β* = −0.396, Adjusted *R*^2^ = 0.427, *ƒ*^2^ = 0.17). Finally, no significant interaction between centered IUS-R and centered P Scale scores emerged, whereas for this step a significant effect for sex emerged (*t* = 2.311, *p* = 0.022, *B* = 3.014, Adjusted *R*^2^ = 0.424).

Pertaining to the second model (T3, April 2020), results are reported in Table 3. The IUS-R emerged as a significant positive predictor of the YSR internalizing problems scale (*t* = 5.837, *p* < 0.001, *β* = 0.45, Adjusted *R*^2^ = 0.202, *ƒ*^2^ = 0.28). The P Scale score then emerged as a significant negative predictor, over and above the effects of the IUS-R (*t* = −3.976, *p* < 0.001, *β* = −0.29, Adjusted *R*^2^ = 0.282, *ƒ*^2^ = 0.092). Finally, and differently from the first model, the interaction between the IUS-R and P Scale emerged to significantly predict the YSR internalizing problems scale (*t* = −3.52, *p* < 0.001, *β* = −0.02, Adjusted *R*^2^ = 0.340, *ƒ*^2^ = 0.06). No significant effect of sex or age emerged for this model.

**Figure 2:**
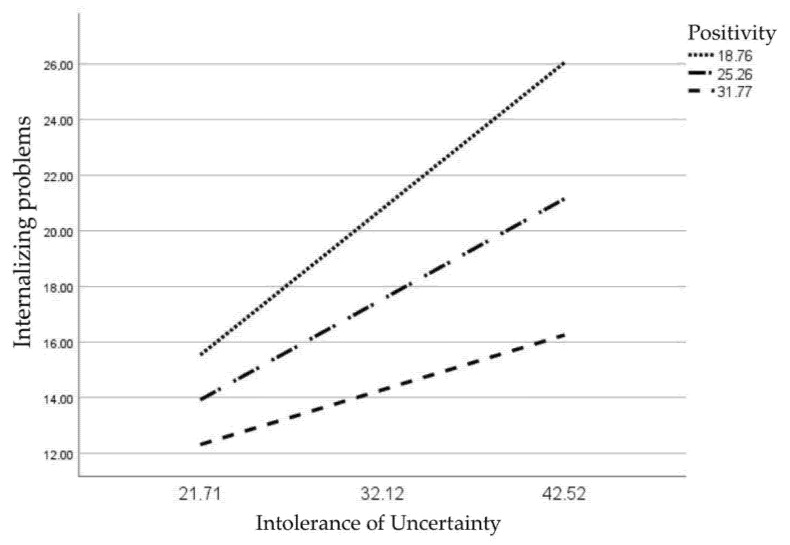
Regression lines of the relation between the IUS-R and the YSR internalizing problems scale scores at high (+1 SD = 18.76), medium (*M* = 25.26), and low scores (−1 SD = 18.76) on the P Scale.

Figure 2 illustrates the interaction at T3 by depicting the regression lines of the relation between the IUS-R and the internalizing problems scale of the YSR, at high, medium, and low (+1 SD, mean, −1 SD) scores of the P Scale. Decreases in the slope of the regression line with increasing P Scale scores show that the relation between IUS-R and internalizing symptoms becomes weaker with higher scores on the P Scale.

The JN technique showed that the conditional effect of the IUS-R on the YSR internalizing problems scale reached significance at a P Scale score of 32.47 (with P Scale scores in our sample ranging between 0 and 38) (*B* = 0.17, *SE* = 0.09, *t* = 1.97, *p* = 0.05, 95% CIs [0.00, 0.34]), at the 90th percentile of the distribution in our sample. Expressly, the relation between IUS-R and internalizing problems was significant at P Scale scores below this threshold and non-significant at P Scale scores above this threshold.

## 4. Discussion

The COVID-19 pandemic has exposed youth to a radical change in environment, involving social isolation and pervasive uncertainty [4]. This picture is further worsened by the fact that adolescence is per se characterized by elevated uncertainty [27] and a heightened vulnerability to psychopathology [14]. However, no research has examined IU and psychopathology in Italian adolescents during the pandemic yet; hence the need for works investigating this topic. In particular, in such a context, it is of the utmost importance that studies focus on risk and protective factors since progress in this line of research might provide healthcare professionals with relevant information in their effort to support young people in these uncertain times, while also contributing to the knowledge surrounding resilience in face of majorly stressful environmental contingencies. In this regard, two well-known risk and protective factors of internalizing outcomes are IU and Positivity.

Bearing all these in mind, the present study aimed to investigate what changes, if any, might have co-occurred with the first COVID-19 lockdown in the psychological well-being and psychopathological symptoms of a group of nonclinical Italian adolescents. Moreover, we sought to explore the unique expression of stable dispositions by investigating the moderating role of a key protective trait (i.e., Positivity) in the relationship between IU and internalizing symptoms before and during the COVID-19 pandemic. Table 4 summarizes which initial hypotheses were confirmed and which were not.

With specific regard to the first objective, no significant changes over time emerged for internalizing, externalizing, and attention problems, generalized anxiety, social anxiety, and psychological well-being. These results seem to describe substantial stability in the mental health of our adolescent sample, in line with our hypotheses (H1a–H1e) and the most relevant literature on these measures during early pandemic months [15,55]. It appears that our group of adolescents showed a fairly good adjustment to the pandemic situation, at least in its first phase. It seems safe to reason that a decreased exposure to an in-person school environment might have led to a reduction in several peer stressors and victimization (for those who experienced it), and academic performance triggers [72,73], which would normally cause distress, anxiety, and depression [74]. The shift to a rather “safe” environment such as the domestic one might not have weighted as much on our sample’s psychological well-being, producing relative stability. Moreover, no significant differences emerged across time points for school and separation anxiety. Presumably, in the early pandemic phase, adolescents still had not come to realize the extent and real significance of this consequential event, and this may have led them to experience it as a break from normal responsibilities. Indeed, it seems fitting to observe that the three measurements are relatively close in time (T1 and T3 are six months apart) and at T3 only two months had passed since the pandemic outbreak. Therefore, it is possible that an early measurement was not able to fully reflect the psychological footprint of the pandemic on Italian adolescents [15,16,17]. Observed under this light, our results would align with the somewhat contradictory evidence [15,16,17], described in the introduction, pointing to a preserved mental health of adolescents during the early months of the pandemic. However, further longitudinal studies are needed to shed light on the long-term consequences of the COVID-19 pandemic on the psychological well-being of youth.

Pertaining to our second aim, we explored the expression of two trait-like dispositions (i.e., IU and Positivity) in two radically different environmental contingencies (i.e., pre-pandemic and during the first national lockdown). In particular, we tested if Positivity had a moderating effect on the relationship between IU and internalizing symptoms and whether the role of Positivity may have changed in the two time points. Findings showed that Positivity significantly moderated the relationship between IU and internalizing problems during the COVID-19 lockdown only, with a small to medium effect size; specifically, the higher an individual’s level of dispositional Positivity, the weaker the relation between IU and internalizing symptoms. These results may suggest that IU is less likely to promote anxiety and depressive symptoms, during a stressful situation, in adolescents with higher levels of Positivity. We speculate that this moderation did not emerge in the pre-pandemic measurement because Positivity might have a resilience-specific nature, waxing in stressful times, but waning in “normal” ones. It is also relevant to note that IU has also been recently conceptualized as a construct that is potentially influenced by situational demands, leading authors to describe it as a trans-situational factor [52,75].

At what levels of Positivity, however, do the protective effects become evident? Probing the interaction between IU and Positivity through the JN technique, we found that the significance of the relation between IU and internalizing problems turned at a Positivity score of 32.47, which within our sample was located at the 90th percentile of the distribution. This value describes how IU exerts its known negative effects on most of our participants and that these effects become entirely offset at relatively high levels of Positivity. As can be seen in Figure 1, these results also tell us that Positivity consistently contributes to the reduction of the negative effect of IU, linearly, across the entire range of values. Summing up, high levels of Positivity fully counteract IU’s negative effect on anxiety and depression, but lower levels linearly contribute to attenuating them, with a small to moderate effect size.

This result is particularly interesting when examined in light of existing literature highlighting the markedly stable nature of these two cognitive components [27,44]. However, it aligns with those studies showing that both Positivity and IU trait expressions are influenced by situational demands [41,42,76]. The literature on Positivity posits that this disposition might represent an influential genetic endowment that can also be subject to modification under the influence of different environmental factors, which account for our position within our personal range of variation at any particular time [39]. Accordingly, it may be that Positivity is a ‘silent’ trait, which increases in weight under uncertain environmental conditions, thus protecting adolescents with high IU levels from developing internalizing symptoms. The striking stability in our psychopathology scores might be read in this light, observing how Positivity, by displaying its malleability, could have protected our adolescent sample from developing a wide range of symptoms. This finding seems to further support the key role played by Positivity in fostering resilience in stressful times [40,45,48,49]. One might speculate that Positivity covaried with IU at T3 because these two cognitive components become tied in the face of uncertain and stressful situations. To some extent Positivity’s weight on IU increased at T3. However, we are not able to dissect this result to determine whether it is specific to the uncertainty component or if it developed a non-specific counteracting effect on other transdiagnostic risk factors. Finally, our results contribute to the growing body of research that signals IU as a risk factor for internalizing symptomatology in adolescence [14].

Concerning the selected control variables (i.e., sex and age) it is worth noting that sex showed a significant predictive effect on internalizing symptoms at T1 only. This result seems at odds with the existing literature [56,57] and suggests a possible transitory interruption in girls’ heightened vulnerability to internalizing symptomatology during the COVID-19 lockdown. As regards the age variable, no effect emerged in either model; this finding is in contrast with our hypothesis (H5) and with a study [58] finding increases in any anxiety and depressive disorder from childhood to adolescence. Generally speaking, these results should be read in light of the specific pandemic phase and its peculiarities, which may have (temporarily) modified some trends in adolescent psychopathology.

Notwithstanding the novel findings that emerged from the present study, some limitations need to be mentioned. Our sample size was reduced in number and limited to only one Italian region which hinders the generalizability of our results. Moreover, the sample is not exhaustive of the adolescent population since we did not benefit from data from early adolescents. Since a clinical sample is missing, we could not examine the exploratory moderation model on individuals suffering from clinical levels of internalizing symptomatology. Future studies should capitalize on these preliminary results to further examine the potential of Positivity in sustaining mental health in at-risk or clinical adolescent samples. Furthermore, an important shortcoming comes from the lack of COVID-19 and lockdown-related control questions addressing key elements such as presence of illness in the family, state of isolation (i.e., quarantine vs. non-quarantined), possible deaths or loss of livelihood in the family, all factors that might have somewhat affected the psychopathological domains addressed in the present study. While the lack of such information does not impact our conclusions, it might have enriched them.

Future research could build upon these initial findings to explore the context-dependent expression of Positivity and IU, both independently and concertedly, in specific transitional phases of adolescence and young adulthood, such as transitions to high school or college [28]. Moreover, the role of Positivity in the relation between IU and anxiety and depressive symptoms could also be explored in the adult population, serving a twofold aim: exploring protective factors to symptomatology during stressful times (i.e., not merely pandemics) and whether our findings are phase-specific to adolescence or can be extended to adulthood.

The present results constitute a novel contribution in that they provide same sample data on early mental health response with baseline pre-pandemic measurements on adolescent mental health during the pandemic. Moreover, to our knowledge, this is the first study to provide data on Positivity in a sample aged 14–18 and to detect a situational effect on Positivity or IU. Such an effort, while explorative in nature, might add to current knowledge on patterns of resilience for these two key constructs, observed in the light of environmentally driven changes, as called for by some authors [39,52]. Additionally, this result appears to tie in with the positive psychology framework, which encourages a shift in focus to how positive and negative factors act concertedly, as opposed to solely on negative factors [31]. Crucially, while these factors were examined under the light of a pandemic outbreak, our results can and should be extended to stressful and uncertain contingencies in general, especially considering the wide range of challenges that adolescents face daily [77].

## 5. Conclusions

In conclusion, this study adds to the extant literature on early mental health response to the COVID-19 pandemic, a large-scale, highly impactful environmental stressor, indicating that our adolescent sample fared well at first. Such a result, along with the literature that supported our hypotheses [15,55] conveys the impression that the initial phase of this pandemic misguided us into observing preserved adolescent psychological functioning. On the contrary, literature addressing later results [78] showed a progressive impairment in adolescent mental health co-occurring with the progression of the COVID-19 pandemic, suggesting that individual protective factors such as Positivity might support psychological well-being, albeit not indefinitely. Additionally, it provides tentative evidence on how two key cognitive contributors to internalizing symptomatology (i.e., Positivity and IU) may act jointly in face of a significant stressor, although further investigations are warranted. Altogether, these considerations support extant literature [79,80] in calling for raised attention towards adolescents’ vulnerable mental health, especially in acute times of uncertainty but also considering the long-term effects of the COVID-19 pandemic. In particular, the observed malleability of Positivity expression hints at the clinical potential of this cognitive construct. As already mentioned, adolescents are under a heightened vulnerability to internalizing problems [14], and uncertainty and IU are elevated in this crucially developmental phase [27]; consequently, interventions aimed at prevention and treatment of such problems might benefit from a focus on positive orientation, which has repeatedly proved critical in supporting resilience, especially during uncertain times.

## Figures and Tables

**Table 1:** Mean values, standard deviations (*SD*), *F*, and *p*-values for the 8 outcome variables (*N* = 136).

	T1 (*SD*)	T2 (*SD*)	T3 (*SD*)	*F*	*p*
YSR Internalizing problems	19.37 (9.23)	18.89 (9.13)	17.54 (9.06)	1.95 (2, 266)	0.145
YSR Externalizing problems	12.76 (7.63)	11.99 (7.05)	11.07 (7.12)	1.87 (2, 266)	0.156
YSR Attention problems	7.18 (3.40)	7.01 (3.38)	6.59 (3.33)	0.87 (2, 266)	0.42
SAFA Generalized anxiety	12.04 (6.49)	11.61 (6.11)	10.99 (5.19)	0.24 (2, 266)	0.78
SAFA Social anxiety	8.92 (4.45)	8.88 (4.97)	8.75 (4.73)	0.122 (2, 266)	0.88
SAFA Separation anxiety	6.71 (4.40)	5.98 (4.45)	6.03 (4.40)	0.39 (2, 266)	0.67
SAFA School anxiety	8.74 (5.27)	8.29 (5.40)	8.26 (5.40)	0.51 (2, 266)	0.60
PWB	52.74 (11.97)	53.79 (13.51)	54.21 (17.40)	0.20 (2, 266)	0.81

Note. YSR: Youth Self-Report; SAFA: Self Administrated Psychiatric Scales for Children and Adolescents-Anxiety evaluation scale; PWB: Psychological Well-Being Scale.

**Table 2:** Results of hierarchical linear regression model examining the main and interactive effects of the IUS-R and P Scale on the YSR internalizing problems scale at T1 (*N* = 136).

				95% CI		
	*β*	SE	*p*	Lower	Upper	Δ*R*^2^
Model 1			<0.001			
Age	0.016	0.623	0.825	−1.094	1.37	
Sex	3.194	1.452	0.03	0.321	6.067	
IUS-R	0.495	0.07	<0.001	0.33	0.606	
Model 2			<0.001			0.148
Age	0.066	0.56	0.32	−0.549	1.668	
Sex	2.959	1.296	0.024	0.395	5.524	
IUS-R	0.420	0.063	<0.001	0.271	0.522	
P Scale	−0.396	0.098	<0.001	−0.774	−0.386	
Model 3			<0.001			0.001
Age	0.56	0.562	0.321	−0.551	1.671	
Sex	3.014	1.304	0.022	0.434	5.593	
IUS-R	0.396	0.064	<0.001	0.27	0.522	
P Scale	−0.584	0.099	<0.001	−0.779	−0.388	
IUS-R × P Scale	−0.006	0.011	0.591	−0.028	0.016	

Note. IUS-R: Intolerance of Uncertainty Scale Revised; P Scale: Positivity scale. Unstandardized coefficients (*B*) are displayed for sex which is a categorical predictor.

**Table 3:** Results of hierarchical linear regression model examining the conditional and interactive effects of the IUS-R and P scale on the YSR internalizing problems scale at T3 (*N* = 136).

				95% CI		
	*β*	SE	*p*	Lower	Upper	Δ*R*^2^
Model 1			<0.001			
Age	−0.002	0.643	0.975	−1.293	1.252	
Sex	2.357	1.484	0.115	−0.578	5.291	
IUS-R	0.449	0.067	<0.001	0.258	0.523	
Model 2			<0.001			0.084
Age	−0.012	0.61	0.874	−1.304	1.110	
Sex	2.082	1.409	0.142	−0.705	4.868	
IUS-R	0.450	0.063	<0.001	0.266	0.517	
P Scale	−0.290	0.102	<0.001	−0.606	−0.203	
Model 3			<0.001			0.06
Age	−0.169	0.586	0.774	−1.327	0.99	
Sex	2.576	1.358	0.06	−0.111	5.263	
IUS-R	0.347	0.062	<0.001	0.225	0.47	
P Scale	−0.501	0.101	<0.001	−0.701	−0.3	
IUS-R × P Scale	−0.024	0.007	<0.001	−0.038	−0.011	

Note. IUS-R: Intolerance of Uncertainty scale Revised; P Scale: Positivity scale. Unstandardized coefficients (*B*) are displayed for sex which is a categorical predictor.

**Table 4:** Hypotheses confirmation or rejection.

Hypotheses	Expected Statistical Significance	Expected Sign	Obtained Sign	Supported/Not Supported
H1a	Not significant			Supported
H1b	Not significant			Supported
H1c	Not significant			Supported
H1d	Not significant			Supported
H1e	Not significant			Supported
H2 at T1	Significant	+	+	Supported
H3 at T1	Significant	−	−	Supported
H4 at T1	Significant	+	+	Supported
H5 at T1	Significant	+		Not supported
H2 at T3	Significant	+	+	Supported
H3 at T3	Significant	−	−	Supported
H4 at T3	Significant	+		Not Supported
H5 at T3	Significant	+		Not supported

## Data Availability

The data presented in this study are available on reasonable request from the corresponding author (S.I.). The data are not publicly available because they report private information about participants.

## Appendix A

**Table A1:** Zero-order correlations for total scores at two time points (T1 and T3).

Time	Variable	1	2	3	4	5	6
Time 1	1. IUS-R	-					
	2. P Scale	−0.199 *	-				
	3. YSR Int.	0.519 ***	−0.479 ***	-			
Time 3	4. IUS-R	0.565 ***	−0.140	0.293 ***	-		
	5. P Scale	−0.142	0.371 ***	−0.326 ***	0.002	-	
	6. YSR Int.	0.408 ***	−0.410 ***	0.603 ***	0.453 ***	−0.295 ***	-

Note. IUS-R = Intolerance of Uncertainty Scale—Revised total score; P Scale = Positivity Scale total score; YSR Int. = Youth Self Report Internalizing problems scale score. * *p* < 0.05; *** *p* < 0.001.
